# Proof of Concept: Game-Based Mobile Learning—The First Experience With the App Actionbound as Case-Based Geocaching in Education of Veterinary Neurology

**DOI:** 10.3389/fvets.2021.753903

**Published:** 2021-12-21

**Authors:** Jasmin Nessler, Elisabeth Schaper, Andrea Tipold

**Affiliations:** ^1^Department of Small Animal Medicine and Surgery, University of Veterinary Medicine Hannover, Foundation, Hannover, Germany; ^2^Centre for E-Learning, Didactics and Educational Research (ZELDA), University of Veterinary Medicine Hannover, Foundation, Hannover, Germany

**Keywords:** bounds, gamification, active learning, teaching, scavenger hunt

## Abstract

Case-based learning is a valuable tool to impart various problem-solving skills in veterinary education and stimulate active learning. Students can solve imaginary cases without the need for contact with real patients. Case-based teaching can be well performed as asynchronous remote-online class. In time of the COVID-19-pandemic, many courses in veterinary education are provided online. Therefore, students report certain fatigue when it comes to desk-based online learning. The app “Actionbound” provides a platform to design digitally interactive scavenger hunts based on global positioning system (GPS)—called “bounds” —in which the teacher can create a case study with an authentic patient *via* narrative elements. This app was designed for multimedia-guided museum or city tours initially. The app offers the opportunity to send the students to different geographic localizations for example in a park or locations on the University campus, like geocaching. In this way, students can walk outdoors while solving the case study. The present article describes the first experience with Actionbound as a tool for mobile game-based and case-orientated learning in veterinary education. Three veterinary neurology cases were designed as bounds for undergraduate students. In the summer term 2020, 42 students from the second to the fourth year of the University of Veterinary Medicine Hannover worked on these three cases, which were solved 88 times in total: Cases 1 and 2 were each played 30 times, and case 3 was played 28 times. Forty-seven bounds were solved from students walking through the forest with GPS, and 41 were managed indoors. After each bound, students evaluated the app and the course *via* a 6-point numerical Likert rating scale (1 = excellent to 6 = unsatisfactory). Students playing the bounds outdoors performed significantly better than students solving the corresponding bound at home in two of the three cases (*p* = 0.01). The large majority of the students rated the course as excellent to good (median 1.35, range 1–4) and would recommend the course to friends (median 1.26, range 1–3). Summarizing, in teaching veterinary neurology Actionbound's game-based character in the context of outdoor activity motivates students, might improve learning, and is highly suitable for case-based learning.

## Introduction

The ability to independently process information and transfer theoretical knowledge for use in a practical setting is the most important ability in veterinary medicine (1). Case-based teaching enhances students' learning engagement *via* the Question–Observation–Doing approach (2). Case-based learning can be used in asynchronous teaching *via* online platforms, for example in CASUS® (3). Here students can solve virtual patient cases at their own speed without the need for contact with real patients. In times of the Coronavirus SARS-CoV-2 (COVID-19) pandemic and subsequent increased amount of online classes, many students were tired of sitting indoor at the desk in front of their computer without contact with their fellow students. In the University of Veterinary Medicine, Foundation, Hannover, an evaluation of the first semester during the COVID-19 pandemic showed that a substantial part of students prefers mobile devices such as smartphones, tablets, or laptops (4) over a desktop computer. The app “Actionbound” provides a platform to design digitally interactive scavenger hunts, called “bounds” and “augments our reality by enhancing peoples' real-life interaction whilst using their smartphones and tablets” (5). It enables the teacher to design bounds in which different tasks are linked to a specific geographic localization within or outside the University campus which needs to be found *via* the global positioning system (GPS) or hints, similar to “geocaching” (6). The designers initially created the app as a multimedia guide for museum tours, city rallies, or team building treasure hunts (5). The app invites the creator of a bound to incorporate videos, photos, hyperlinks to websites, multiple-choice questions (MCQ), and a variety of different challenges. Students can score with the right answers or solve different tasks. In the present study, we combined case-based teaching with the app Actionbound and created three cases on the base of real clinical patients in the field of veterinary neurology. We aim to present the app “Actionbound” as a tool for mobile game-based learning in veterinary education; show how a scavenger hunt can be used as a platform for case-based learning; and evaluate the student acceptance of this learning format.

## Methods

To design three bounds for case-based learning, the app Actionbound (Actionbound GmbH, Hohenpeißenberg, Germany) was used for veterinary students. To use the app as an educational institution, a custom license for universities was used. Students can use the app for free. Preexisting data of real canine patients of the Department of Small Animal Medicine and Surgery of the University of Veterinary Medicine, Foundation, Hannover, were used to create the bounds. The cases cover three of the most common problems in veterinary neurology: a Labrador Retriever with seizures, a Boxer with paraparesis, and an Australian Shepherd with tetraparesis (1).

**Figure 1:**
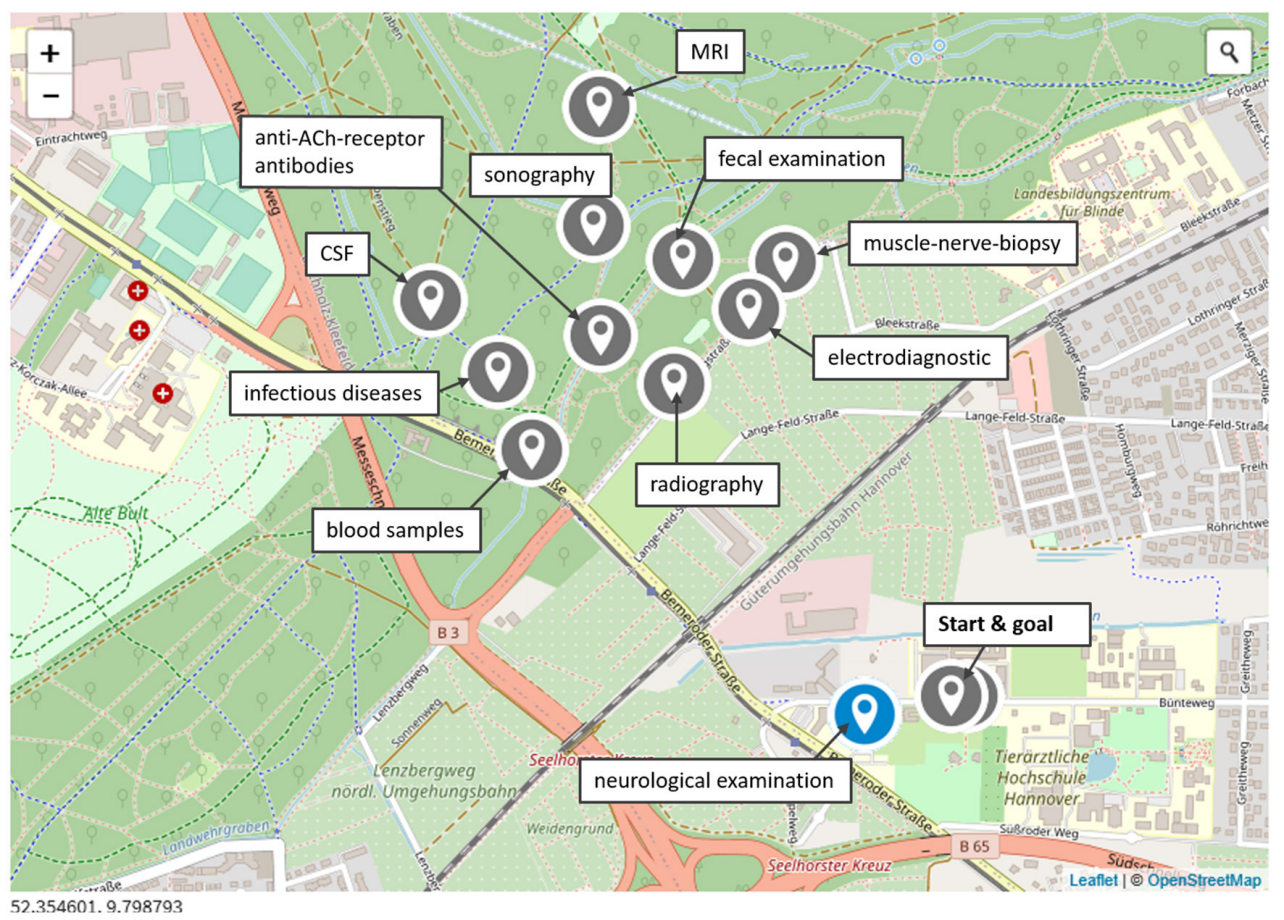
Map. Case of the tetraparetic Australian Shepherd. Map of Hannover, Germany. Every section of the bound is connected with a single examination and guides the students *via* the global positioning system (GPS) to another geographical localization on the campus and the surrounding forest. MRI, magnetic resonance imaging; CSF, cerebrospinal fluid; ACh, acetylcholine. Modified from Actionbound (6).

Each bound consists of three parts. The first part includes an introduction and the case vignette and consists of 5–16 screens. The second part is divided into several sections. Every case contains 5–10 sections, each including one examination or test, like neurological examination, radiographs, blood examination, cerebrospinal fluid examination, or other different advanced techniques necessary to find a diagnosis for the patient. In the second part, students can choose which examination they want to perform and can choose their sections accordingly (Table 1). Every section consists of 2–9 screens with tasks, questions, or explanations. Every section guides the students *via* GPS to another geographical localization on the campus and the surrounding forest (Figure 1).

**Table 1:** Different sections in each bound.

	**Labrador Retriever with seizures**	**Paraparetic Boxer**	**Tetraparetic Australian Shepherd**
**The start** mandatory	Case vignette	Case vignette, general clinical examination, neurological examination	Case vignette, general clinical examination, neurological examination
**The examinations** students can select examinations and the order they want to study them	General clinical examination	Radiography	Radiography
	Neurological examination	Computed tomography, myelography	Blood examination
	Radiography	Magnetic resonance imaging	Abdominal ultrasonography
	Blood examination	Cerebrospinal fluid examination	Fecal examination
	Abdominal ultrasonography	Blood examination	Cerebrospinal fluid examination
	Cardiac ultrasonography		Magnetic resonance imaging
	Advanced diagnostic imaging		Electrodiagnostic examination
	Cerebrospinal fluid examination		Muscle and nerve biopsy
			Anti-ACh-receptor antibody test
			Infectious agents
**The end** mandatory	Diagnosis, prognosis, therapy, outcome	Diagnosis, prognosis, therapy, outcome	Diagnosis, prognosis, therapy, outcome

*Despite a mandatory start-and-end section, every case contains 5–10 sections, each including one examination or test, necessary to find a diagnosis for the patient, which can be chosen independently*.

*ACh, acetylcholine*.

The walking distance between every section is about 200–500 m. Questions and tasks can be answered if the students are within a 10-m radius of the given localization. The third and last parts of the bounds include the summary of the results of the preceded examinations and the diagnosis, prognosis, treatment, and outcome of the case. When a bound is created, Actionbound provides a quick response (QR) code, which can be passed to the students, to scan and start the bound. Actionbound provides several question-and-answer formats: multiple-response questions (single- or multiple-answer format), single best-answer questions, free text answers, estimation of numbers, or creative answer formats such as uploading of audios or videos. Using the last format, students have to record their answer or film movement/neurologic examination of themselves or of their dogs. Correct answers to MCQ and free text answers were rewarded with points. Depending on the difficulty and the importance of the question, 50–500 points could be gained. Creative audio, photo, or video answers were not graded.

**Figure 2:**
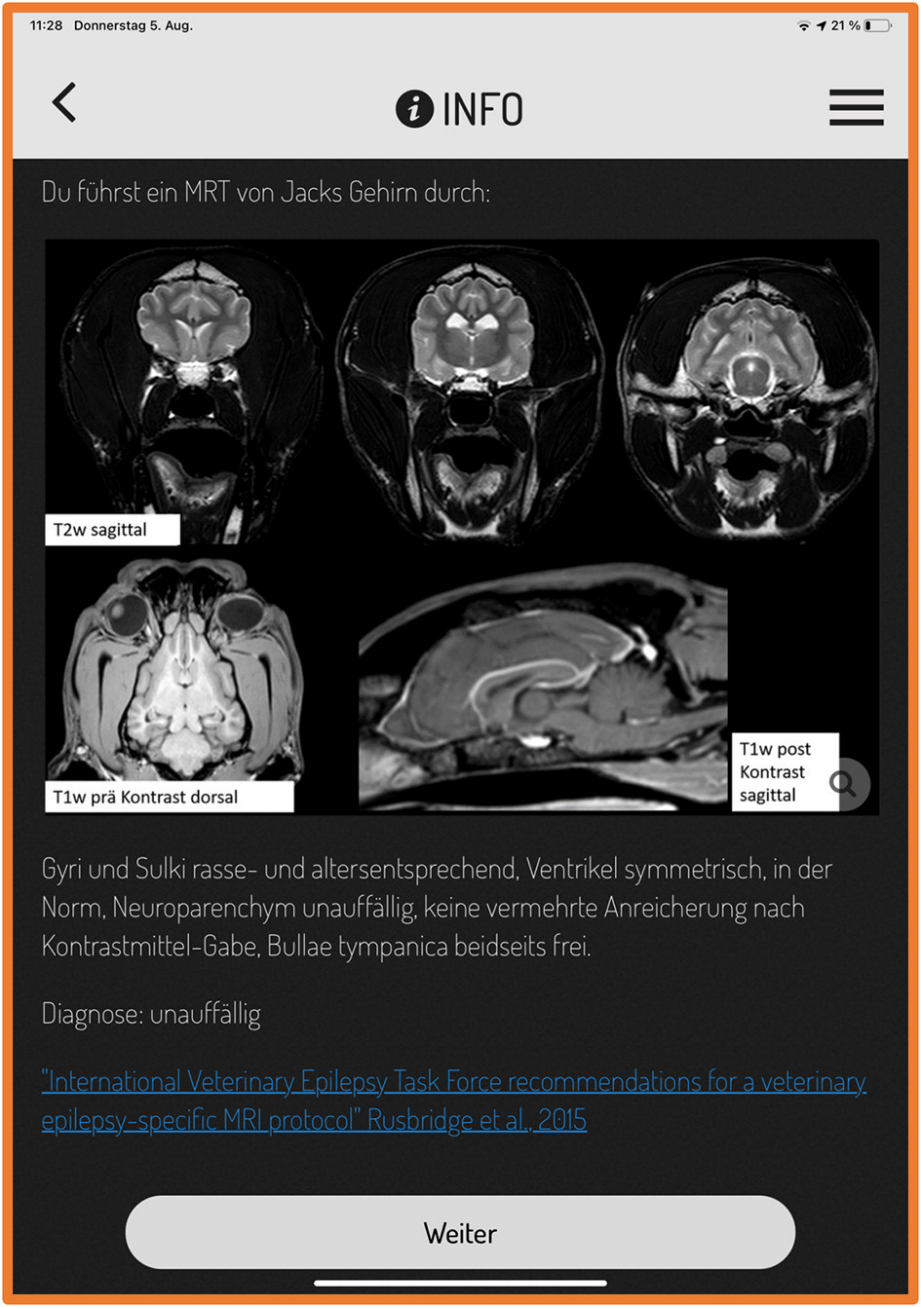
Original screenshot of actionbound. The screen displays the magnetic resonance imaging (MRI) results of a Labrador Retriever with seizures and a link to an international consensus statement about epilepsy-specific MRI protocols. (Text says: “You performed a MRI of Jack's brain:”; “T2w sagittal”; “T1w pre contrast dorsal”; “T1w post contrast sagittal”, “Gyri and sulci breed and age specific normal. Ventricles symmetrical, normal, brain parenchyma unremarkable, no pathological contrast uptake, tympanic bulla both sides filled with air.”; “next”). T2w, T2 weighted; T1w, T1 weighted.

In the course of the bound, different information were provided to the students like scientific literature on the subject, instructions on how to perform an examination, results of the neurological examination, or radiography. Therefore, several media were used: text, videos, photos, or hyperlinks to websites (Figure 2). To demonstrate how to perform a diagnostic test—for example, cerebrospinal fluid examination—links to learning videos on the University's YouTube channel [https://www.youtube.com/user/TiHoVideos; (7)] were provided. The details and results of the clinical examinations and diagnostic tests were given *via* text, photo, or video of a real patient, depending on what was appropriate for the particular test.

Veterinary students from the second to the fourth year could enroll in the elective course called “clinical neurology on the move” in the summer term 2020. The course encompassed periods, where the students could solve a bound at any preferred time within a timeframe of 4 weeks, alternating with synchronous online classes *via* MS Teams® (Microsoft, Redmond, WA, USA), where the opportunity was offered for the students to ask questions and get help with technical or professional questions. A new bound was provided every 4 weeks. The students were instructed to prepare for each case with selected book chapters, PowerPoint presentations, or peer-reviewed articles. The learning material and the QR code to start the bound were provided *via* the online platform MS Teams®.

Students were allowed to play each bound alone or could form teams of two or three students. They could start the bound whenever they wanted within a given time period of 4 weeks. After downloading the app Actionbound from the app store on their smartphone, they opened the app and scanned the given QR code starting the bound. At the beginning of the bound, students had to register themselves as single player or as a team. Every case started in front of the Department of Small Animal Medicine and Surgery, where students have access to free university's WLAN to pre-load the media, which is not mandatory but recommended by the app. Subsequently, the students moved on the university's campus and the surrounding forest and searched for GPS coordinates where they gain information on examinations or solve questions and tasks (Figure 1). Due to the current pandemic situation, some of the students preferred to stay in their hometowns. Therefore, for every case, an additional “corona-home-edition bound” was created, which was identical to the normal bound, but finding the localization *via* GPS was excluded.

**Figure 3:**
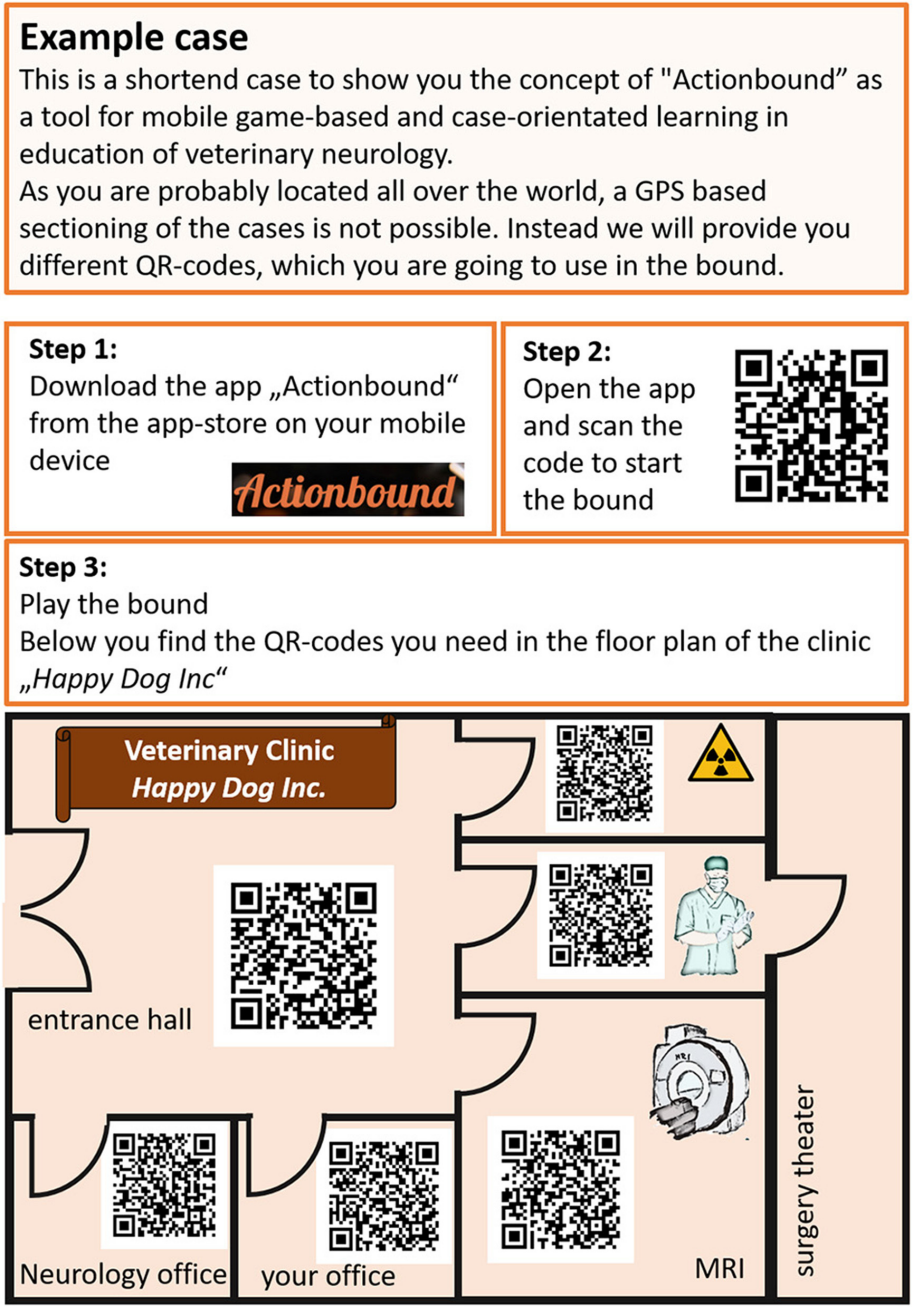
Example case. Follow the instructions to play an example case, which is a short adapted English version of an original bound (paraparetic Boxer). GPS, global positioning system; QR code, quick response code.

For every case, students were stepwise led to recognize pathological findings and name them. The bounds were designed that students could define the problem by creating a problem list, specifying and naming the main problem [“diagnostic hook;” (8)], assigning the main problem to an organ system, and specifying the clinical problem. Then, the students created a list with likely differential diagnoses. Subsequently, they were supposed to choose the different sections according to the examinations they wanted to “perform” to find the final diagnosis. In every section, questions and tasks were included to repeat and transfer previous theoretical knowledge necessary to understand and evaluate the results of the diagnostic test. To play an example case, see Figure 3.

Chosen examinations, answers, and time to completion of the bound were recorded by the app for every played bound per team or per single player. All recorded video and audio answers given by the students can be retrieved by the creator of the app on the Actionbound homepage in the log in area and were evaluated for the study. Scores were recorded as total points and as the percentage of total possible points for each bound.

At the end of each bound, students evaluated the bound *via* questionnaire. Thus, the students filled in the same questionnaire once after each solved bound. They were asked to evaluate different aspects of the course and the app with a 6-point numerical Likert rating scale [1 = excellent, 2 = good, 3 = satisfactory, 4 = adequate, 5 = poor, 6 = unsatisfactory; (Table 2)]. Additionally, different questions with free text answers were given (“What did you like the most?”, “What did you dislike?” and “How can the course be improved?”).

**Table 2:** Likert rating scale of the evaluation questionnaire.

**Please rate**	**1**	**2**	**3**	**4**	**5**	**6**
The app in general						
Reaction time of the app						
GPS connection						
Time to download videos						
Time to download photos						
Videos and photos were well visible						
Walking distance						
Duration of the bound						
Difficulty of the questions						
The course in general						
Would you recommend this course to your friends?						

*After each bound, students evaluated different aspects of the course and the app with a 6-point numerical Likert rating scale (1 = excellent, 2 = good, 3 = satisfactory, 4 = adequate, 5 = poor, 6 = unsatisfactory)*.

*GPS, global positioning system*.

Statistical analysis and comparison of time to completion and scores between groups (indoor bound vs. outdoor bound; teams vs. single players; fourth-year students vs. second- and third-year students) were performed for every case in indoor and outdoor versions separately using the t-test with SAS Enterprise Guide 7.15 (SAS Institute Inc., Cary, NC, USA). p values are given in the results or in the tables. p values (*p* < 0.05) are considered significant.

This study was conducted according to the ethical standards of the University of Veterinary Medicine Hannover, Foundation. The data protection officer reviewed and approved the proposed project regarding observance of the data protection law. All the data obtained were processed and evaluated anonymously and in compliance with EU's General Data Protection Regulation.

## Results

In the elective course, 42 students were enrolled. One student was in the second year, 21 in the third year, and 20 in the fourth year. In three bounds, it was unclear which students had played the bounds, as their names were not clearly identifiable. Twenty-one students played all three bounds, 17 students solved two bounds, and four students played only one bound. Students played a total of 88 bounds: Case 1 and case 2 were each played 30 times, and case 3 was played 28 times (Table 3). The outdoor version of the bounds was played 47, and 41 times the corona-home edition was used. The students solved 60 bounds as teams by two or three; 28 bounds were solved by single players. Teams mostly played the outdoor versions (*n* = 42/60 teams), while the corona-home editions were solved by single players most often (*n* = 23/28 single players).

**Table 3:** Amount of solved bounds.

	**Bounds solved**	**Total**	**Outdoor walking = GPS based**	**Home edition = indoor without GPS**
**Case 1**	Labrador Retriever with seizures	*n =* 30	*n =* 17	*n =* 13
**Case 2**	Paraparetic boxer	*n =* 30	*n =* 17	*n =* 13
**Case 3**	Tetraparetic Australian shepherd	*n =* 28	*n =* 13	*n =* 15
**Total**		***n =*** **88**	***n =*** **47**	***n =*** **41**

*Students could play every case as one bound as single player or in a team*.

*GPS, global positioning system; n, number*.

The students took 59.2–89.7 min (12–238 min, *n* = 39) on average to solve the corona-home edition of the bounds, and 117.9–161.6 min (31–307 min; *n* = 47) to solve the outdoor bounds (Table 4). Two teams were excluded here from the evaluation, because of technical errors, which prevented them from finishing the bounds on time. There was no significant difference in time between teams or players of the fourth, third, or second year (*p* = 0.06–0.8, *n* = 86 bounds). The students achieved on average 59.49% (18.41–98.37%; *n* = 88 bounds) of possible points. In the majority of bounds, teams or single players of the fourth study year achieved slightly more points than teams or single players of the third year, but this was not significant (Table 5). Students playing the outdoor bounds achieved more points than students who played the corona-home-editions when case 1 or 2 was played (Table 6). However, there was no significant difference if students played in a team or as single player (*p* = 0.1–1).

**Table 4:** Time to complete bound.

**Bounds solved**		**Outdoor walking = GPS based**	**Home edition = indoor without GPS**
		**mean (min-max)** **[minutes]**	**mean (min-max)** **[minutes]**
**Case 1**	Labrador Retriever with seizures	161.7 (93–221)	89.7 (33–238)
		*n =* 17	*n =* 11
**Case 2**	Paraparetic Boxer	161.2 (31–307)	59.2 (12–145)
		*n =* 17	*n =* 13
**Case 3**	Tetraparetic Australian Shepherd	117.9 (77–183)	82.9 (18–225)
		*n =* 13	*n =* 15

*Two teams were excluded here from the evaluation, because of technical errors, which prevented them from finishing the bounds on time*.

*GPS, global positioning system; min, minimum; max, maximum; n, number*.

**Table 5:** Points.

	**Third year**	**Fourth year**	
	**mean (min-max)** **[% of possible points]**	**mean (min-max)** **[% of possible points]**	
**Case 1 Labrador Retriever with seizures**			
Outdoor walking = GPS based	60.77 (40–88.91) *n =* 6	64.01 (46.53–88.91) *n =* 9	*p =* 0.7
Home edition = indoor without GPS	38.98 (18.41–58.76) *n =* 6	53.55 (20.02–81.63) *n =* 7	*p =* 0.2
**Case 2 paraparetic Boxer**			
Outdoor walking = GPS based	63.47 (42.92–84.70) *n =* 6	66.58 (51.25–92.95) *n =* 11	*p =* 0.7
Home edition = indoor without GPS	46.11 (38.62–62.73) *n =* 6	58.16 (46.39–83.04) *n =* 7	*p =* 0.07
**Case 3 tetraparetic Australian Shepherd**			
Outdoor walking = GPS based	60.46 (53.13–67.38) *n =* 5	66.72 (49.43–80.44) *n =* 7	*p =* 0.3
Home edition = indoor without GPS	61.43 (42.54–98.37) *n =* 8	58.61 (40.49–75.29) *n =* 7	*p =* 0.7

*Percentage of possible points every team or single player gained in a bound; teams might be mixed with students of different years, here the “oldest” student in the team is counted; p < 0.05 is considered significant. In three bounds the study year of the students was unknown and were therefore excluded from the calculation*.

*GPS, Global Positioning System; min, minimum; max, maximum; n, number*.

**Table 6:** Points.

	**Bounds solved**	**Outdoor walking = GPS based**	**Home edition = indoor without GPS**	
		**mean (min-max)** **[% of possible points]**	**mean (min-max)** **[% of possible points]**	
**Case 1**	Labrador Retriever with seizures	62.83 (40–88.91) *n =* 17	46.83 (18.41–81.63) *n =* 13	*p* = 0.01
**Case 2**	Paraparetic Boxer	65.48 (42.92–92.95) *n =* 17	52.60 (38.62–83.04) *n =* 13	*p* = 0.01
**Case 3**	Tetraparetic Australian Shepherd	63.83 (49.43–80.44) *n =* 13	60.11 (40.49–98.37) *n =* 15	*p* = 0.4

*Percentage of possible points every team or single player gained in a bound*.

*GPS, global positioning system; min, minimum; max, maximum; n, number*.

Questionnaires for 88 bounds were returned, including incomplete questionnaires. The biggest part of the students graded the course as excellent to good (median 1.35, 1–4; *n* = 86 answered questions) and would recommend the course to friends (median 1.26, 1–3; *n* = 86 answered question).

Students evaluated the app in general with a median score of 1.35 (1–3; *n* = 85 answered questions), the reaction time of the app with 1.5 (1–3; *n* = 86 answered question), GPS connection 1.92 (1–3; *n* = 77 answered questions), and time to download videos and photos with 1.4 (1–3; *n* = 84 answered questions), respectively, with 1.34 (1–3; *n* = 83 answered questions). Pictures (including radiographs, pictures of magnetic resonance imaging, and computed tomographic imaging) were well visible for the majority of students (median 1.69, 1–4; *n* = 87 answered questions).

The walking distance for each bound was approximately 3–5 km. The distance and duration of the bounds were graded as excellent to satisfactory by most students (length: median 2.24, 1–5; *n* = 66 answered questions // duration: median 2.14, 1–5; *n* = 85 answered questions). Twelve of 86 students felt that the questions were too difficult for them (median 2.45, 1–5), of which four students were in the fourth year.

In the free text answers of the evaluation questionnaire, the majority of students highlighted the outdoor activity, fresh air, and exercise. Many students enjoyed the alternation to pure screen-based learning and thought the bounds to be exciting and fun. Many students took their dogs with them for a walk and even included them in their video answers, although this was no explicit task. Most students, who performed both a corona-home version and an outdoor version, preferred being outside in a team over studying at home alone. Only one student preferred the corona-home edition, as this person had better possibilities to make notes at home. Some students criticized that the given route was not a clear circuit but led in a zigzag through the forest or was too long. They proposed a preview showing the potential route before starting the bounds. Most students reported it was easy to understand the usage of the app. Technical errors occurred rarely.

Students stated in the free text answers of the evaluation questionnaire that they preferred multiple-choice questions. In Actionbound, free text answers count as false if the spelling is incorrect and small spelling mistakes led to loss of points, although the answer was correct from a professional point of view. This demotivated most of the students. Many students indicated in the free text answers of the evaluation questionnaire that they got also demotivated, if in multiple-choice questions with multiple right answers the full amount of points was lost when only a part of all correct answers was chosen. Students proposed to give points proportionally, which is possible in Actionbound.

**Figure 4:**
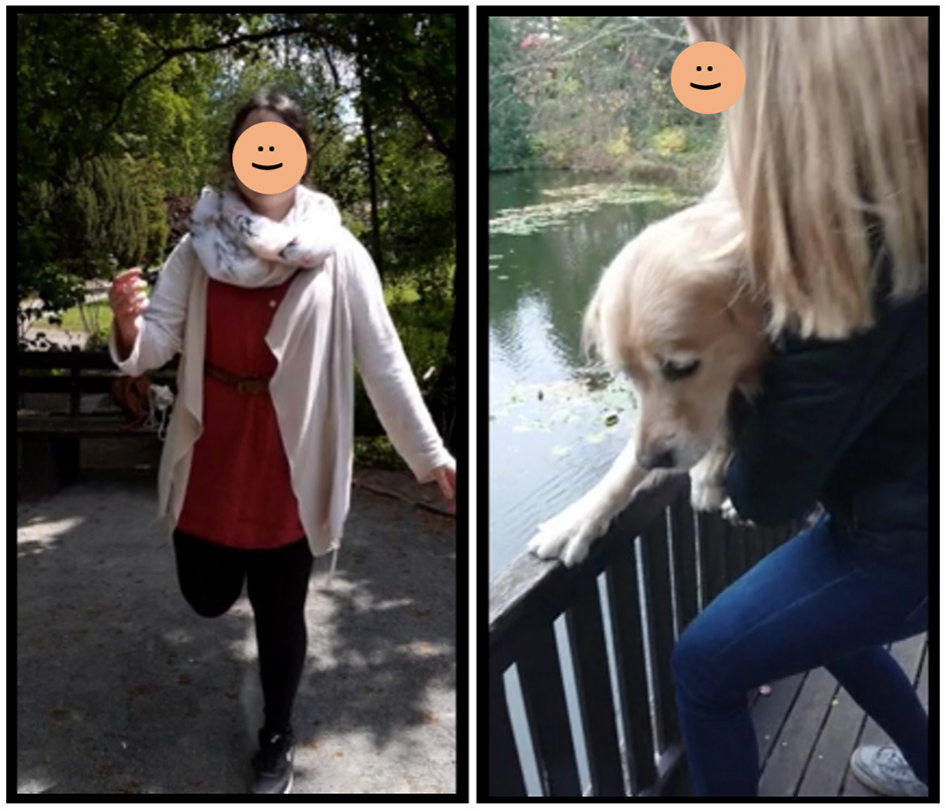
Screenshot of video answers. Both screenshots are part of video answers from students. They answered the question “Proprioception means the ability to sense the position of the body and the limbs. Record a short video (10–20 s) where you show how proprioception can be tested.” The first picture shows a student while balancing on one leg. The second picture displays a student performing the placing reaction with her own dog.

In the Actionbound app, students were able to deliver technical answers in a creative way *via* video and audio answers (Figure 4). When the videos were reviewed by the authors on the Actionbound homepage, most students seemed to have fun and laugh on the videos, although some students indicated in the free text of the evaluation questionnaire that they feel uncomfortable recording themselves.

Most students appreciated the practical and case-based aspects of learning, as well as the intense and specific engagement with one disease. Some liked the independent learning and the given material to prepare, while others had problems understanding the scientific articles or the questions in the app. Some students had trouble deciding which examinations made sense for their case. On the other hand, some of them admitted they did not prepare with the given materials before they played the bounds.

Creating one case lasts for the teacher approximately 6–10 working hours. If problems appear, detailed help is available *via* the help forum (https://forum.actionbound.com/c/english-support).

## Discussion

In case-based teaching, the teacher presents a clinical problem and asks the students to identify the problem and figure out a solution within the learning process. It combines different disciplines and invites the student to decide priorities and choose their individual approach to solve the clinical problem (9). For the students, this adds practical relevance and meaning to the theoretical subject matter. It requires active learning, which is defined as instructional activities which involve students in doing and thinking about what they are doing. Such activities can only be performed, when higher-order thinking is applied (10). Active connection of already existing knowledge and new experiences form and enhance deeper understanding (11). This context-driven approach and the deep insight into a field foster personal development and enhance intrinsic motivation of students (11). This study presents the mobile app Actionbound as a tool that can be used for case-based learning in veterinary medicine. The app allows creating a GPS-based scavenger hunt with narrative elements, quizzes, and different tasks. Bounds on the base of real veterinary patients repurposed Actionbound from a multimedia guide for museum tours into a veterinary case-based geocaching and made it an exciting opportunity that allowed veterinary students to literally search examinations and test results *via* GPS on the campus and in the forest to get a diagnosis. Several teaching methods are combined here: gamified classical case-based learning, mobile learning, and active outdoor exercise.

Students imbibed the possibility of gamification. Actionbound enhances learning engagement as it provides immediate rewards to the students in form of scores for correct answers. This prompt positive feedback acts as a reward and motivates to continue in the learning process (12, 13). On the contrary, students got demotivated if they felt that they have been treated unfairly; e.g., small spelling mistakes led to loss of points, although the answer was correct from a professional point of view. However, Actionbound offers the opportunity of “relaxed scoring” where partial points might be given for every correct answer.

For a positive game and learning experience, technical components might be important. The students indicated in the questionnaire that they could download the pictures and videos in a reasonable timeframe and were satisfied with several technical aspects of the app: despite small displays of smartphones and possible sunlight reflections on the screen for most students, all pictures of, e.g., radiographs and magnetic resonance imaging, were well visible according to the answers in the questionnaire. It might be important to pay attention to the quality of the chosen pictures, so they can be well visible even on small screens. Minor insecurities occurred in using the app in the very first bound. Technical training is recommended to get to know the course and the app. It might be reasonable to design an introduction bound that does not include any medical questions, to get the students used to the handling of the app (14). Technical errors of the software or the hardware might impair the learning experience, and not every student might have a suitable mobile device. Here, universities might provide mobile devices if needed.

Cases for the bounds were chosen to impart knowledge on the most important learning objectives in veterinary neurology, as defined in a survey among general veterinary practitioners and European specialists for veterinary neurology (1): paraparesis (intervertebral disc herniation), tetraparesis (polyneuropathy), and seizures. The app is highly suitable to impart the steps of Bloom's taxonomy of learning, which are “remember,” “understand,” “apply,” “analyze,” and “do” (15). MCQs may assess simple remembered facts and ask for understanding of formerly processed literature. Similar to conventional case-based education methods, the students need to apply factual knowledge throughout the whole bound (16). As an example, students have to choose the appropriate clinical or special examinations. The advantage of Actionbound compared to other case-based computer programs used at our University (e.g., CASUS®) might be that the student can choose the examination in his/her individual order depending on his/her individual preferences (3). Therefore, this feature is even closer to real-life scenarios. Analyzing the given clinical findings and drawing a conclusion requires a high amount of conceptual and procedural knowledge (15). On the other hand, mobile learning is not suitable to communicate complex topics or high amount of information (17). Although Actionbound allows providing whole manuscripts and articles, readability might be impaired due to a small screen or reflections of sunlight on the screen, although students did not report this issue in the evaluation questionnaire. Complex content needs concentration (17), and in-depth research might benefit from a calm and low-stimulus environment and a desk where the students can take notes easily. This was mentioned by one student who preferred playing the bounds indoors at a desk.

Actionbound offers the possibility to design the case studies in a way that different examinations are linked to a specific geographic localization within or outside the campus which needs to be found *via* GPS, similar as geocaching does. The bounds were designed to lead the students in a campus-near city forest where they solved a route of 2–4 km. To increase the context sensitivity, one could even use the advantage to include a contextual location into the learning process and design the route of the bound, e.g., within the Clinic for Small Animals (17). Nevertheless, students' evaluation of the Actionbound course confirmed the aspect of walking outside and inhaling fresh air as one of the most valuable aspects of the bounds. When designing the route of the bound, it might be important to consider the requirements of physically disabled students and provide an indoor version as performed in the current study.

Mostly, students who played outdoor achieved significantly more points than students who played indoor at home. It is known that exercise may enhance memory encoding (18). Additionally, autobiographical experiences, which is part of episodic memory, enhance the retrieval of semantic memory, which was encoded in this special learning experience (19). Because of the experience-oriented approach, Actionbound might enhance episodic memory encoding much more intensely for education as the uniform experience of primary desk-based online teaching can do. Actionbound enables breaks between each bound station, where students move from one localization to the next without any learning stimuli. This corresponds with studies about spaced learning theory which found that long-term memory encoding was improved and faster, because of short periods of highly compressed instructions alternating with spaces of 10-min distractor activities (20).

Learning experience and students' motivation might depend on weather conditions. Bad weather might limit students' motivation to spend time outdoors for a bound. Therefore, an indoor alternative might be needed, e.g., a non-GPS-based indoor variant as shown in the present study.

In the case of the Labrador Retriever with seizures and the paraparetic Boxer, students performing the bounds outdoors gained significantly more points than students who worked on the corresponding indoor bound. Teams of students were more likely to play outdoor bounds and could benefit from the knowledge of the other team members. On the other hand, no difference could be found in the number of achieved points if students played as a single player or in a team. To confirm that outdoor activity in case-based learning can enhance the learning performance compared to case-based learning in front of the desktop, comparative studies have to be performed. Additionally, it needs to be addressed if case-based learning with Actionbound is superior to other conventional teaching methods or is only a suitable supplement to deepen clinical knowledge and understanding. Having such additional information would be a prerequisite to include this tool in the routine veterinary curriculum. The app does not allow direct synchronous communication with the teacher for theoretical questions. Therefore, for the implementation in the curriculum, an additional alternative platform would be necessary to communicate with students to solve questions subsequent to the bound.

Students enjoyed the team work during the bounds. Active learning often involves group work (21). The described bounds were played in small teams to create a peer-to-peer interaction. The cognitive and social congruence creates a nonjudgmental learning atmosphere (22) and drives discussion as well as fosters personal development (11).

## Conclusion

Actionbound is a feasible tool to offer unconventional case-based learning *via* GPS-based scavenger hunts to veterinary students suitable to increase motivation of students. Currently, this tool seems to be a good supplement to desk-based approaches of case-based learning and other conventional learning methods. For implementation in a curriculum, comparative studies with conventional teaching methods are needed.

To help students starting this app and to reduce initial hesitancy of taking electives with a new learning approach, an introduction bound to explain the handling of the app and the idea behind Actionbound case-based learning is recommended. An additional platform should be offered for synchronous help and further communication.

Summarizing, the present proof of concept confirmed that case-based geocaching is accepted well by students, a well-perceived elective and supplementation to traditional teaching methods. Most students highlighted the outdoor activity, fresh air, and exercise. Actionbound as case-based geocaching stands out as an appealing gamification tool for veterinary education: with frisky outdoor activity, it can motivate students to address a clinical problem in depth.

## Data Availability Statement

The raw data supporting the conclusions of this article will be made available by the authors, without undue reservation.

## Author Contributions

JN drafted the study design, designed the bounds, collected the data, and drafted and wrote the article. ES drafted the study design and added valuable comments to the article. AT drafted the study design and drafted and finalized the article. All authors contributed to the article and approved the submitted version.

## Funding

This publication was supported by Deutsche Forschungsgemeinschaft and University of Veterinary Medicine Hannover, Foundation, within the funding program Open Access Publishing.

## Conflict of Interest

The authors declare that the research was conducted in the absence of any commercial or financial relationships that could be construed as a potential conflict of interest.

## Publisher's Note

All claims expressed in this article are solely those of the authors and do not necessarily represent those of their affiliated organizations, or those of the publisher, the editors and the reviewers. Any product that may be evaluated in this article, or claim that may be made by its manufacturer, is not guaranteed or endorsed by the publisher.
